# Rapid Diagnosis of Malaria

**DOI:** 10.1155/2009/415953

**Published:** 2009-06-11

**Authors:** Clinton K. Murray, Jason W. Bennett

**Affiliations:** Infectious Disease Service, Brooke Army Medical Center, 3851 Roger Brooke Drive, Fort Sam Houston, TX 78234, USA

## Abstract

Malaria's global impact is expansive and includes the extremes of the healthcare system ranging from international travelers returning to nonendemic regions with tertiary referral medical care to residents in hyperendemic regions without access to medical care. Implementation of prompt and accurate diagnosis is needed to curb the expanding global impact of malaria associated with ever-increasing antimalarial drug resistance. Traditionally, malaria is diagnosed using clinical criteria and/or light microscopy even though both strategies are clearly inadequate in many healthcare settings. Hand held immunochromatographic rapid diagnostic tests (RDTs) have been recognized as an ideal alternative method for diagnosing malaria. Numerous malaria RDTs have been developed and are widely available; however, an assortment of issues related to these products have become apparent. This review provides a summary of RDT including effectiveness and strategies to select the ideal RDT in varying healthcare settings.

## 1. Introduction

According to the World Malaria Report released by the World Health Organization (WHO) in 2008, there were 247 million malaria cases among 3.3 billion people at risk in 2006 from 109 countries resulting in an estimated 881,000 deaths. These deaths were primarily in Africa (91%) and in children under 5 years of age (85%) [1]. Effective management of malaria per the WHO has focused on long-lasting insecticidal nets, indoor residual spraying of insecticide, intermittent preventive therapy in pregnancy, and artemisinin-based combination therapy (ACT). Fundamental to improving the care of patients infected with malaria is prompt and accurate diagnosis in order to prevent excess morbidity and mortality while avoiding unnecessary use of antimalarial agents and minimizing the spread of resistance to antimalarial drugs. Diagnostic strategies need to be effective not only in resource-limited areas where malaria has a substantial burden on society but also in developed countries where expertise in the diagnosis of malaria is frequently lacking [2, 3]. 

Historical strategies to diagnose malaria emphasize clinical diagnostic algorithms, light microscopy, and empiric therapy. The accuracy of clinical diagnosis, the most commonly employed method, is poor, even in countries with high incidence rates of malaria due to overlapping clinical symptoms with other tropical diseases and the fact that coinfections can occur [4–11]. In an era when chloroquine therapy was widely effective and the primary antimalarial agent used around the world, definitive diagnosis was not necessary. Today, given the paucity of effective antimalarial agents with widespread use of ACT, an increased emphasis on diagnosis is necessary. This is especially true now that artesunate failures have been noted [12]. Microscopy remains the gold standard for detection of malaria parasitemia as it can provide information on both the species of parasite and parasite density of infection [13]. However, the procedure is labor-intensive and time-consuming, requiring substantial training and expertise due to fleeting skills [14–26]. These problems are magnified in nonendemic regions where light microscopy to diagnose malaria is infrequently performed, resulting in missed diagnosis, misidentification of *Plasmodium* species, and therapeutic delays [19]. Methods using advances in technology have been evaluated as alternatives to light microscopy. While these methods have varying strengths and weaknesses, they are limited by equipment, supplies, expertise, cost, time, applicability in acute infection, and/or availability [13, 27]. 

New immunochromatographic rapid diagnostic tests (RDTs) for malaria were introduced in the early 1990s but have suffered from numerous problems as recently reviewed [13, 27–31]. Strategies to improve the impact of malaria RDT have included The Special Programme for Research and Training in Tropical Diseases which has introduced principles for development and evaluation of diagnostic tests for infectious diseases [32]. The WHO has also undertaken a malaria RDT evaluation program to improve the overall impact of these tests (http://www.wpro.who.int/sites/rdt/who_rdt_evaluation/ accessed 19 January 2009).

Requirements for malaria RDT vary based on malaria local epidemiology and the goals of a malaria control program; focusing on performance and operational characteristics (Table 1) [33, 34]. Expectations of an ideal malaria RDT are minimal operator training, ease of platform with minimal steps, reproducibility of results, rapid availability of results (<20 minutes), and low cost. Ideally, the test should be able to detect recrudescence or relapse and clearance of parasitemia if therapeutic monitoring is necessary (Table 1). The WHO has recommended a minimum standard of 95% sensitivity at parasite densities of 100/uL [33]. In Sub-Saharan Africa, malaria RDT needs a high sensitivity for *Plasmodium falciparum*, but specificity is required to avert inflated estimates of the burden of malaria, misperceptions of inadequate therapeutic responses when fever is due to other illnesses, and unnecessary drug pressure. High specificity and the ability to detect *nonfalciparum* *Plasmodium* species are necessary in low-incidence regions due to low rates of malaria, and the lower virulence of these species is allowing for repeat testing. The quality of manufacturing and reproducibility of the test results are critical. In addition, field conditions require the RDT to be stable under extremes of temperatures and humidity during use and storage.

## 2. Malaria Rapid Diagnostic Tests

Malaria RDT employ lateral flow immunochromatographic technology similar to rapid pregnancy tests. In these assays, the clinical sample migrates as a liquid across the surface of a nitrocellulose membrane by capillary action [13, 27, 33, 34]. For a given targeted parasite antigen, two sets of monoclonal or polyclonal antibodies are used, a capture antibody and a detection antibody. Monoclonal antibodies in contrast to polyclonal antibodies can be very specific but less sensitive. Also, the source of antigen used (purified native protein, recombinant proteins, or peptides) can make significant differences in the performance characteristics of RDT. 

The malaria antigens currently used as diagnostic targets are either specific to a *Plasmodium* species or are conserved across all four of the human malaria parasites. Falciparum-specific monoclonals include histidine-rich protein-2 (HRP-2) and *P. falciparum* lactate dehydrogenase (pfLDH) [32, 33]. Targets conserved across all human malaria have been identified on lactate dehydrogenase (pLDH) and aldolase enzymes [35–38]. 

HRP-2 is a *P. falciparum*-specific water-soluble protein, localized in the parasite cytoplasm and on the surface membrane of infected erythrocyte. It is present on protrusions, known as knobs, thought to account for sequestration of the trophozoites and schizonts in postcapillary venules. There is increasing concentration of HRP-2 as the parasite advances from ring stage to trophozoite, and it readily diffuses into the plasma [39, 40]. HRP-2 is predominately found in the asexual stages, but it is also found in young *P. falciparum* gametocytes. This possibly allows detection at lower parasitemias and at detectable levels 28 days after clinical presentation, well after resolution of symptoms and apparent clearance of parasites from patients [41–44]. Therefore, this antigen has not yet proven valuable in monitoring response to therapy. Mutants can escape recognition by monoclonal antibodies and may be responsible for false negative tests [45, 46]. An assessment of HRP-2 from nineteen countries revealed that only 84% of *P. falciparum* could be detected. 

Another antigen target to detect sexual and asexual stage malaria parasites is Plasmodium lactose dehydrogenase (pLDH), which is the final enzyme in the malaria parasite's glycolytic pathway. Monoclonal antibodies against pLDH can target all human malaria species or can specifically differentiate *P. falciparum* or *P. vivax* [36]. Aldolase, another key enzyme in the glycosis pathway conserved across all malaria parasites, can be used as a universal antigen target [47, 48]. Other antigens have been recognized as possible components of future diagnostic tests, but evaluations of *P. ovale*- or *P. malariae*-specific antigens have not been widely tested [38, 49, 50]. Aldolase and pLDH rapidly fall to undetectable levels after initiation of effective therapy, however they are expressed in gametocytes, as does HRP-2, which may allow detection of *P. falciparum* after the clinical infection is cleared [51].

### 2.1. Available Malaria Rapid Diagnostic Tests

Numerous reviews have highlighted the rapid turnover of commercially available products and varying quality control issues in manufacture and product stability [13, 27–29, 52]. Articles in peer-reviewed journals of independent evaluations have not existed for many products. In addition, there are numerous methodological flaws associated with many of the published evaluations limiting the ability to compare malaria RDT [13, 27]. The WHO has listed online RDT manufacturers and distributors. To be included in these summaries requires evidence of good manufacturing practices as documented by either compliance with ISO 13485:2003 or 21 CFR 820 from the US Food and Drug Administration. Overall it appears that RDTs using HRP-2 are generally more sensitive than falciparum-specific pLDH for diagnosing infections caused by *P. falciparum* when using RDT. However, data assessing the utility of pLDH and aldolase in nonfalciparum infections is limited.

Currently the BinaxNOW^®^ Malaria test kit is the only US FDA approved kit. It is based upon the HRP-2 and aldolase antigens (Binax, INC., Inverness Medical Professional Diagnostic, Scarborough, Me, USA). One large trial revealed an overall sensitivity of 82% [44]. The overall specificity for *P. falciparum* was 94% [44]. Again, using the BinaxNOW Malaria test kit, a second trial primarily assessed the utility of finger-stick versus venipuncture obtained blood samples [53]. The finger-stick technique revealed an overall sensitivity of 100% for *P. falciparum* and 83% for *P. vivax*. Venipuncture produced similar results to fingerstick for the detection of *P. falciparum* and *P. vivax* (Table 2). According to the package insert, the overall sensitivity and specificity are 95% and 94% for *P. falciparum*, 69% and 100% for *P. vivax*, respectively (Table 2). The sensitivity for detecting *P. malariae* was 44% (7 of 16 positive samples) and 50% for *P. ovale* (1 of 2 positive samples), although these numbers were too small to determine reliable sensitivity and specificity. Although the kit is not approved for diagnosing mixed *P. falciparum* and *P. vivax* infections, the sensitivity was 94%. No clear data exists for using RDT to detect a recently described species of malaria, *P. knowlesi*, that has been reported to infect humans [54]. 

Overall, the BinaxNOW^®^ Malaria test kit meets many of the needs required of an ideal malaria diagnostic platform; however, it has also a number of limitations including the FDA indications comment that this kit is only for use in laboratories that have or can acquire blood samples containing *P. falciparum* for use as a positive control. It is also approved for use in the evaluation of symptomatic patients, with negative results requiring confirmation by thick and thin smears. Therefore individual clinicians or patients themselves might not have rapid results to initiate immediate therapy. Other limitations include the kit's ability to detect viable and nonviable malaria organisms, including gametocytes and sequestered *P. falciparum* parasites. In some settings, such as pregnancy, this is possibly advantageous; however, this prohibits monitoring the level of parasitemia, which is often used in management decisions. Additionally this prevents the monitoring of therapeutic response as antigens persist after elimination of the parasite. Finally, positive rheumatoid factor has been associated with false positive results. Overall, many of these limitations also plague other RDT [13, 27].

### 2.2. Applicability of Malaria Rapid Diagnostic Tests

It has been estimated that 16 million RDTs were delivered in 2006 of which 10.8 million were in Africa and 2.8 million in India [1]. Malaria RDTs are used at almost every level of the healthcare system. Most of the data supports using these devices in settings where trained personnel perform the assay in targeted adult patient populations presenting with a febrile illness. There is limited data in children [44]. Among pregnant women, *P. falciparum* malaria is associated with placental sequestration of parasites that can reduce the sensitivity of microscopic diagnosis. In this clinical scenario, the detection of HRP-2 might improve diagnostic capability as this antigen is recovered peripherally [55]. However, the relevance of persistent HRP-2 antigen for up to a month after therapy is unclear in this setting. Overall, the ideal setting for these devices would include use by village workers without formal medical laboratory training, or travelers for self-diagnosis and treatment; however, there is conflicting evidence on the utility of RDT in these settings [56–59]. The diagnosis of *P. falciparum* has been made on convalescent serologic (day 14–21 after febrile illness) or post-mortem assessments, all supporting possible diagnosis after initiation of empiric therapy [13, 60].

Other possible indications for malaria RDT could include malaria prevalence surveys; however, they are insensitive for use in asymptomatic screening and high throughput detection cannot be achieved with individually packaged RDT [17, 61–64]. Currently the American Red Cross does not screen blood donation units for malaria, instead deferring donations based upon exposure risks.

### 2.3. Selection of Malaria Rapid Diagnostic Tests

Selection of specific malaria RDT is based upon region specific criteria including the expected health benefit, implementation plans, monitoring process, and cost with a focus on expected species of infection, level of parasitemia, and treatment paradigms. Parasitological confirmation of the diagnosis of malaria is recommended in all cases except for children under 5 years of age residing in areas of high prevalence of *P. falciparum*. It is unclear if the risk of not treating false-negative tests outweighs the benefits of empiric therapy. 

The WHO has typically outlined 3 broad zones for selecting devices. Zone 1 occurs primarily in Sub-Saharan Africa and in lowland Papua New Guinea where infections occur with *P. falciparum* only or where nonfalciparum species occur as coinfections with *P. falciparum*. The HRP-2-based kits are probably best in this region because of overall improved antigen detection for *P. falciparum*. Zone 2 occurs in endemic areas of Asia, the Americas, and in isolated areas in Africa specifically the Ethiopian highlands, where falciparum and nonfalciparum malaria typically cocirculate. RDT in these regions will need to distinguish between falciparum and nonfalciparum infections. Zone 3 contains areas with nonfalciparum malaria only; including the *P. vivax* areas of East Asia and Central America. Here, RDT should focus on *P. vivax-*specific or pan-Plasmodium specific antigen detection without a need to detect or differentiate falciparum. Even in *P. falciparum* predominate regions, it is possible for 1–10% of patients to be coinfected including cases requiring anti-relapse therapy with primaquine [65]. 

Although the major burden of malaria is in endemic countries, malarious regions are frequent destinations of the roughly 900 million yearly international travelers (www.unwto.org/index.php, accessed 24 January 2009). These travelers might require management of their malaria infection while abroad or upon returning home; however, laboratory personnel in nonendemic regions often lack experience or expertise in microscopic diagnosis of malaria [66]. Based upon a large meta-analysis of malaria RDT use in travelers, RDT may be an effective adjunct to microscopy in centers without substantial expertise in tropical medicine [52, 67]. However, expert microscopy is still needed for species identification and confirmation. Strategies need to be developed to determine how best to evaluate and field malaria RDT for use in nonendemic regions and for travelers who will be on holiday for prolonged periods of time in highly endemic regions.

## 3. Conclusions

Malaria is a life-threatening infection impacting the most developed countries of the world along with regions of the world lacking basic healthcare infrastructure. Increasing burden of disease, emerging antimalarial drug resistance, and broad implementation of ACT are placing greater emphasis on rapid and accurate diagnosis of patients infected with malaria. Given the difficulty performing microscopy, especially in endemic areas, alternative diagnostic strategies are needed. A highly effective RDT could avert over 100.000 malaria related deaths and about 400 million unnecessary treatments [68]. In addition, it is likely that RDTs will be cost-effective due to improved treatment and health outcomes for febrile disease not due to malaria along with cost savings associated with antimalarial drugs [69]. Although there is now an FDA approved malaria RDT, RDTs have limitations to include the inability to detect mixed infections, all species of *Plasmodium*, and infections at low concentrations of parasites, along with an inability to monitor response to therapy. In addition, in the case of a negative result, microscopy is still recommended. Therefore RDTs do not eliminate the need to obtain thick and thin smears, and maintaining expertise in microscopy is still a global priority until a new gold-standard is developed. However, malaria RDTs are ushering in a new era of diagnosis to improve the overall global healthcare system. 

## Disclaimer

The opinions or assertions contained herein are the private views of the authors and are not to be construed as official or reflecting the views of the US Department of the Army, the US Department of Defense, or the US government. The authors are employees of the US government and this work was performed as part of their official duties. As such, there is no copyright to be transferred.

## Figures and Tables

**Table 1:** Ideal requirements for a malaria rapid diagnostic test (RDT).

Common requirements			
Rapid results (<20 minutes)			
Easy to use with minimal training and simple instructions			
Environmentally stable device (heat, humidity, air movement, lighting) during use and storage			
Reproducible results including quality manufacturing			
Detect below parasite density of <100 parasites/*μ*L with appropriate specificity			

Region-specific requirements			

	Sub-Saharan Africa	Other malaria endemic areas	Malaria free countries

***P. falciparum-specific***	+++	−	−
***Detects all human malaria***	+	+++	+++
***Plasmodium species specific***	+	+++	+++
***Able to detect mixed infections***	+	+++	+++
***High sensitivity (***<***50 parasites/*** *μ* ***L)***	−	+++	+++
***High specificity***	−	+++	+++
***Semi-quantitative***	−	−	+++
***Able to monitor response to therapy***	+	+++ (if drug-resistant *P. falciparum*)	+

***Assay specifications:***			

***ICH GMP***	−	−	+++
***Stable to 40°C***	+++	+++	−
***Long shelf life***	+++	+++	+++
***Point-of-care use (CLIA waived)***	−	−	+++
***Cost***	<$1 per test	$1–3 per test	<microscopy (~$20/test)

+++ (high priority), + (low priority), − (not necessary), International Conference on Harmonization (ICH); Good Manufacturing Practices (GMP); Clinical Laboratory Improvement Amendment (CLIA).

**Table 2:** Performance characteristics of the BinaxNOW^®^ malaria kit for *Plasmodium falciparum* and *P. vivax *(data obtained from package insert)*.*

	*Plasmodium falciparum*	*Plasmodium vivas*
Parasitemia level (per *υ*L)	Percent sensitivity (95% confidence interval)	Percent sensitivity (95% confidence interval) %)

>5000	99.7% (98%–100%)	93.5% (91%–96%)
1000–5000	99.2% (96%–100%)	81.0% (76%–85%)
500–1000	92.6% (76%–99%)	47.4% (36%–59%)
100–500	89.2% (75%–97%)	23.6% (17%–31%)
0–100	53.9% (37%–70%)	6.2% (3%–12%)
Overall	95.3% (93%–97%)	68.9% (66%–72%)
Specificity	94.2% (93%–95%)	99.8% (99%–100%)

Venous versus fingerstick samples	100% (96–100%) versus 98.8% (94–100%)	81.6% (74–87%) versus 80.6% (73–87%)
Specificity	94.7% (93–96%) versus 90.4% (88-92%)	99.7% (99–100%) versus 99.5% (99–100%)
